# Inquiries Respecting the Efficacy of the Oil of Turpentine in the Treatment of Neuralgia, and Particularly of Sciatica

**Published:** 1829-03

**Authors:** M. Martinet


### OIL OF TURPENTINE.

*Inquiries respecting the Efficacy of the Oil of Turpentine in the Treatment of Neuralgia, and particularly of Sciatica.* By M. MARTINET.

TURPENTINE was employed many ages ago in the treatment of diseases of the nerves. It was used by Galen and Michael Doringius in the form of a plaster; Scultet exhibited it successfully in wounds of the nerves; and Bonnet had even the good fortune to cure a patient of neuralgia by the essential oil of turpentine: but Archibald first brought it into notice as a remedy for sciatica. Having informed Cheyne of the success with which he had employed it in this disease, the latter recommended it to Home, who afterwards published, in his "Experiments and Facts," seven cases in confirmation of the practice. Since that period turpentine has been employed in the above-mentioned diseases by many physicians of different countries: by Helst, Thilenius, and Lentin, in Germany; and by Recamier, De Larroque, Dufour, and Husson, in France.

M. Martinet affirms that little benefit is to be expected from the employment of the oil of turpentine, without due attention to the mode of administering it. It has been exhibited in various proportions, and in very different ways, but he decidedly prefers giving it internally, and in small doses of twenty drops three times daily, in order that its absorption may be the more gradually but thoroughly effected. In larger doses it is liable to occasion diarrhœa, by which its peculiar properties are rendered unavailing. The oil of turpentine, when thus given in scruple doses, and in some proper vehicle, such as honey, syrup, or (what is still better) in magnesia, by which its acrid taste is more completely disguised, produces a strong sensation of heat in the stomach and whole intestinal tube, as well as in the diseased nerve and limb; and sometimes it even occasions a general sweat. In certain individuals it causes a slight colic or a mild diarrhœa, and, more rarely, either a dysury or an increased flow of urine. But when a drachm of the medicine is taken instead of a scruple, intense colic, diarrhœa, and even vomiting, supervene; yet these formidable signs of irritation, both of the digestive and the urinary organs, generally disappear as soon as the medicine is intermitted. In patients whose stomach and bowels are irritable, a small quantity of opium is found a useful addition to the turpentine, by moderating its stimulating effects on the mucous membrane of those parts.

When the oil of turpentine is administered in the manner and quantity just described, it would seem to be particularly powerful in the removal of sciatica; yet, as M. Martinet suggests, this opinion may have arisen from the greater frequency of this complaint. Be this as it may, its efficacy is also remarkable for curing other species of neuralgia which affect the extremities.

When we attempt to deduce from the phænomena which follow the exhibition of the oil, the mode of its operation, and the cause of its being efficacious, we cannot refer the latter either to its purgative, its diuretic, or its sudorific effects; since this augmentation of the different secretions is neither regular nor constant in its occurrence, and never bears any proportion to the benefit derived by the patient. Besides, we daily see patients who are purged, sweated, &c. much more abundantly by other medicines, without deriving the least benefit; and it was this reflection which led Home to attribute to the oil of turpentine a specific influence over sciatica.

Some physicians have supposed that this medicine produces its sanitary effects on the nervous system by causing a revulsion from the brain to the stomach and skin; but M. Martinet thinks he has clearly shown in his Essay that these effects are almost always missing, even in cases of recovery: and he, therefore, will not admit this explanation to be correct. Others, on the contrary, attribute its efficacy to a revulsion on the nerves which is sympathetic with that of the stomach.

M. Martinet, however, conceives that the stimulation which this oil communicates to the mucous membrane of the stomach is equally produced in the nerves affected, and to a greater or lesser degree, in proportion as they are more or less morbidly affected; which, in his opinion, serves to explain the fact that this medicine is more efficacious in severe and obstinate, than in mild and recent cases of neuralgia. The new modification which is thus effected in the state of the nerve would seem, therefore, to dispose it to resume its natural action, that of health. The heat which the greater proportion of those persons who are either cured or relieved feel in the affected parts, seems to confirm the explanation adopted.

As to the question whether the turpentine acts directly on the nerves by absorption, or exerts its influence over them indirectly and sympathetically, through the medium of the stomach, we are most inclined to adopt the first of these hypotheses: and we found our opinion on the fact that this oil is nearly always observed to fail in curing those cases of neuralgia where it produces violent purging; which is also true in respect to all other substances employed in this disease, and whose only effect is to irritate the mucous membrane of the stomach and intestines. Its action on the urinary organs would appear to be seldom useful, but, on the contrary, often injurious.

As an external remedy, turpentine seems most beneficial when rubbed in by the hand: it thus produces redness of the surface, without exciting a sensation of heat along the course of the nerve. But the strong and penetrating odour of the oil, when exhibited in this manner, not unfrequently occasions headach.

This medicine is of approved efficacy in all cases of neuralgia affecting the extremities, and particularly in sciatica, when this disease is simple in its character, and evinces no sign that the nerve is either altered in its structure, in a state of inflammation, or compressed by the formation of a contiguous tumor. M. Martinet affirms that, whether the complaint be recent or otherwise, the chance of cure by this remedy is greatest, *cæteris paribus*, when the pain is so intense as to indicate distinctly the course of the nerve, and so obstinate in its nature as to yield to no other treatment whatever. But it is necessary to pay attention to the state of the stomach; for, in case it should not be perfectly sound, the medicine must be immediately intermitted.

Twelve days usually suffice for curing neuralgia when it affects the extremities, and, more commonly, only half that time; and the exhibition of this remedy during a longer period would therefore be injudicious, and detrimental to the organs of digestion.

That the reader may be enabled to judge for himself respecting the correctness of the doctrines above advanced, we shall terminate the present paper by giving a brief analysis of the various observations which M. Martinet has included in his Essay.

Of seventy individuals affected chiefly with sciatica, and other kinds of neuralgia of the extremities, fifty-eight were cured: viz. three by rubbing in the oil, and all the others by taking it internally; ten, two of whom prematurely intermitted the medicine, obtained only temporary relief from its use; and five received no benefit. Of these five, two had diseases of the joints, of which they died a few months afterwards.

Of these seventy-one cases of neuralgia, (for one of the patients had two affections of the kind,) forty were acute, and thirty-one chronic. Of the forty acute cases, thirty-four were cured, five relieved, and only one continued in the same state. Of the thirty-one chronic cases twenty-four were cured, three relieved, and four received no amendment.

Again, of the seventy-one cases of neuralgia, thirty-three had resisted the effects of divers remedies; and, of these thirty-three, twenty-five were completely cured, four were only relieved, and four more remained uninfluenced by the medicine.

Of the fifty-eight cases of neuralgia which were cured by the oil of turpentine, thirty-four were cured in less than ten days; twenty-two in less than twelve days; and three within the space of from twenty-eight to forty-five days.

Of the same fifty-eight cases which were cured, forty-eight were cases of sciatica, two of which were cured by frictions; three were cases of crural, four of brachial, and three of facial neuralgia.

Of the ten patients which were only relieved, two were affected with sciatica, and their treatment was intermitted on the second day.

Finally, of the five in which the treatment entirely failed, there were four cases of sciatica, and one of crural neuralgia. Two of the four died of disease of the hip-joint.

In twenty-one instances it is recorded that a sensation of heat was experienced along the course of the nerve, and in the affected limb; and of these, nineteen were perfectly cured; the other two, having intermitted the treatment, were only relieved.

In eighteen cases a sensation of heat was felt in the intestines, and especially in the stomach. Three were seized with vomiting, in two of whom it was occasioned by a too powerful dose of the oil, namely, two drachms.

Three suffered from diarrhœa and severe colics; and in one instance the inside of the patient's mouth was affected with vesicles.

In five cases the urine was more abundant than natural; and four were attacked with strangury. Two of these had taken too large a dose.

In ten patients there was sweating over the whole body, and in two instances it was confined to the member affected.

And, lastly, one woman, affected with neuralgia, was, as it were, intoxicated by the turpentine; and two other patients experienced the sensation of itching throughout the whole body.

